# Anti-Müllerian hormone levels and live birth rate in polycystic ovary syndrome patients undergoing assisted reproductive technology: a systematic review and meta-analysis

**DOI:** 10.3389/fmed.2026.1859573

**Published:** 2026-07-28

**Authors:** Xiangui Wei, Yuqing Wang, Tingting Hu, Hua Li, Ling Liu, Zhaohui Li, Xiaoli Zhao

**Affiliations:** 1 Integrated Traditional Chinese and Western Medicine Reproductive Center, Hohhot First Hospital, Hohhot, China; 2 Department of Obstetrics and Gynecology, Chifeng Municipal Hospital, Chifeng, Inner Mongolia Autonomous Region, China; 3 Reproductive Center, First Teaching Hospital of Tianjin University of Traditional Chinese Medicine, Tianjin, China

**Keywords:** anti-Müllerian hormone, polycystic ovary syndrome, live birth rate, clinical pregnancy rate, assisted reproductive technology

## Abstract

**Background:**

Anti-Müllerian hormone (AMH) is typically elevated in women with polycystic ovary syndrome (PCOS), but its relationship with assisted reproductive technology (ART) outcomes remains unclear.

**Objectives:**

This systematic review and meta-analysis aimed to evaluate the association between AMH levels and pregnancy outcomes in women with PCOS undergoing ART.

**Search methods:**

Observational studies published between January 2020 and May 2025 were identified from major databases. The search strategy incorporated controlled vocabulary and keywords related to “Polycystic Ovary Syndrome,” “anti-Müllerian hormone,” and ART outcomes. Eight studies were included.

**Main outcomes:**

Higher oocyte yields were observed across ascending AMH subgroups, with significant differences detected in all pairwise comparisons (low vs. high: MD = −3.91, 95% CI: −5.60 to −2.23; Z = 4.55, *P* < 0.001, I^2^ = 86%; low vs. medium: MD = −2.33, 95% CI: −2.79 to −1.86; Z = 9.77, *P* < 0.001, I^2^ = 50%; medium vs. high: MD = −1.70, 95% CI: −3.31 to −0.09; Z = 2.07, *P* = 0.04, I^2^ = 0%). Higher AMH levels were significantly associated with increased risk of ovarian hyperstimulation syndrome (OHSS). Compared with the low AMH group, the high AMH group had a higher OHSS risk (OR = 0.64, 95% CI: 0.47–0.88, Z = 2.77, *P* = 0.006, I^2^ = 0%). Similarly, the high AMH group also presented a significantly elevated OHSS risk relative to the medium AMH group (OR = 0.40, 95% CI: 0.23–0.70, Z = 3.19, *P* = 0.006, I^2^ = 0%). Paradoxically, reproductive outcomes appear more favorable in participants with lower AMH levels: low AMH was associated with higher live birth rates versus both medium (RR = 1.15, 95% CI: 1.07–1.24; Z = 3.71, < 0.001, I^2^ = 39%) and high AMH (RR = 1.19, 95% CI: 1.10–1.28; Z = 4.53, *P* < 0.001, I^2^ = 27%), as well as a lower miscarriage risk (low vs. medium: RR = 0.60, 95% CI: 0.42–0.86; Z = 2.80, *P* = 0.005, I^2^ = 0%; low vs. high: RR = 0.72, 95% CI: 0.53–0.98; Z = 2.10, *P* = 0.04, I^2^ = 49%). No significant associations were found between AMH levels and cumulative live birth rates, clinical pregnancy rates, or number of good-quality embryos.

**Conclusion:**

The distinct intermediate phenotype of the medium AMH group, exhibiting superior live birth outcomes compared to high AMH, yet inferior to low AMH, underscores the value of finer stratification beyond a simple binary classification.

**Systematic review registration:**

https://www.crd.york.ac.uk/prospero/, identifier [CRD420251089070].

## 1 Introduction

Polycystic ovary syndrome (PCOS) is a prevalent endocrine disorder affecting women of reproductive age, with an estimated prevalence of 8%–20% and is characterized by ovulatory dysfunction and hyperandrogenism as core clinical features (1–3). Amidst rapid lifestyle and societal shifts, the incidence of PCOS has risen markedly, posing serious threats to female fertility, quality of life, and long-term health. This escalating disease burden represents a critical public health challenge that requires urgent intervention.

For patients with PCOS-related infertility, although ovulation induction therapy can restore ovulatory function in the majority of patients, the cumulative pregnancy rate over three treatment cycles ranges from 17.8% to 42%, with a high miscarriage rate of 33.8% (4, 5). Even with in vitro fertilization-embryo transfer (IVF-ET) technology, live birth rates for PCOS patients typically range from 23.3% to 44% (6).

Anti-Müllerian hormone (AMH), secreted by granulosa cells of preantral and small antral follicles, is a direct serum indicator of ovarian antral follicle reserve. Some studies have demonstrated that low AMH levels (<3.7 ng/mL) are associated with improved oocyte maturation and higher pregnancy rates, whereas elevated AMH levels (>7.4 ng/mL) may be a prognostic indicator for poorer IVF outcomes in women with PCOS (7). Some reports indicate that although individuals with high AMH concentrations (12–25 ng/mL) yield a significantly greater number of retrieved oocytes compared to those with low AMH (1–12 ng/mL), this increase does not translate into significant differences in pregnancy or live birth rates (8). Recent evidence further suggests no significant differences in clinical pregnancy, live birth, or miscarriage rates between PCOS patients with high AMH (>9.3 ng/mL) and those with low AMH (≤9.3 ng/mL) (9). Conversely, other studies have reported comparable pregnancy rates between high (>8.27 ng/mL) and low (<3.32 ng/mL) AMH groups, but a significantly reduced live birth rate among women with elevated AMH (10).

## 2 Materials and methods

This systematic review and meta-analysis adhered to the Preferred Reporting Items for Systematic Reviews and Meta-Analysis (PRISMA). The protocol was prospectively registered with PROSPERO (ID number: CRD420251089070).

### 2.1 Search strategy and selection criteria

A systematic literature search was performed in PubMed, Web of Science, Embase, and the Cochrane Library to identify relevant English-language studies examining PCOS, anti-Müllerian hormone (AMH), and assisted reproductive outcomes published between January 2020 and May 2025 (we restricted publication year to post-2020 to minimize extra heterogeneity from outdated AMH testing standards and inconsistent controlled ovarian hyperstimulation protocols adopted in earlier published researches). Search terms included controlled vocabulary (e.g., MeSH headings) and free-text keywords such as “Polycystic Ovary Syndrome,” “Anti-Müllerian Hormone,” “Ovarian Response,” “Ovulation Induction,” and “Reproductive Outcomes.” Boolean operators were employed to combine conceptual domains. Additionally, reference lists of included articles were manually screened to identify potentially eligible studies. Detailed search terms for all databases are provided in Supplementary Table 1.

Search results were imported into EndNote and subjected to primary screening based on predefined inclusion and exclusion criteria. Two independent reviewers (Yuqing Wang and Xiaoli Zhao) evaluated titles and abstracts for eligibility. Discrepancies were resolved through consultation with a senior researcher or by consensus discussion.

Inclusion criteria encompassed observational investigations (cohort, case-control, prospective or retrospective designs) of reproductive-aged PCOS patients undergoing ART. Eligible studies were required to report pre-treatment serum AMH levels and present at least one of following ART outcomes: live birth rate (LBR), cumulative live birth rate (CLBR), clinical pregnancy rate, miscarriage rate, number of oocytes retrieved, or incidence of ovarian hyperstimulation syndrome (OHSS), stratified by AMH level. We excluded meta-analyses, reviews, conference proceedings, editorials, comments, letters and practice guidelines, studies with incomplete data, and those from which relevant data could not be extracted.

### 2.2 Data extraction and assessment of risk of bias

Two review authors (Yuqing Wang and Xiangui Wei) independently extracted outcome data from eligible studies, and one review author (Yuqing Wang) entered the data into RevMan software. The methodological quality of the eight included studies was assessed using the Newcastle-Ottawa Scale (NOS). This tool employs distinct versions for cohort and case-control studies, both structured around three core domains: (1) selection; (2) comparability; (3) outcomes assessment (for cohort studies) or exposure ascertainment (for case-control studies). Each version contains eight items assessed through a star-based rating system, with a maximum possible score of nine stars for both study designs.

### 2.3 Outcomes and exposures

The primary outcome was live birth rate. Secondary outcomes included clinical pregnancy rate, miscarriage rate, number of oocytes retrieved, good-quality embryo count, and incidence of OHSS.

### 2.4 Data analysis

Statistical analyses were performed using Review Manager (RevMan, version 5.4) and Stata (version 14.0). Heterogeneity was evaluated with Cochran’s Q test and quantified using the I^2^ statistic. A random-effects model (DerSimonian-Laird method) was employed when substantial heterogeneity existed (I^2^ > 50%); otherwise, a fixed-effects model (Mantel-Haenszel method) was applied. Publication bias was planned to be assessed through funnel plot symmetry inspection and Egger’s regression test (when ≥ 10 eligible studies were available). Sensitivity analyses were conducted by sequential exclusion of individual studies where significant heterogeneity (I^2^ > 50%) persisted.

## 3 Results

### 3.1 Literature search and studies characteristics

**FIGURE 1:**
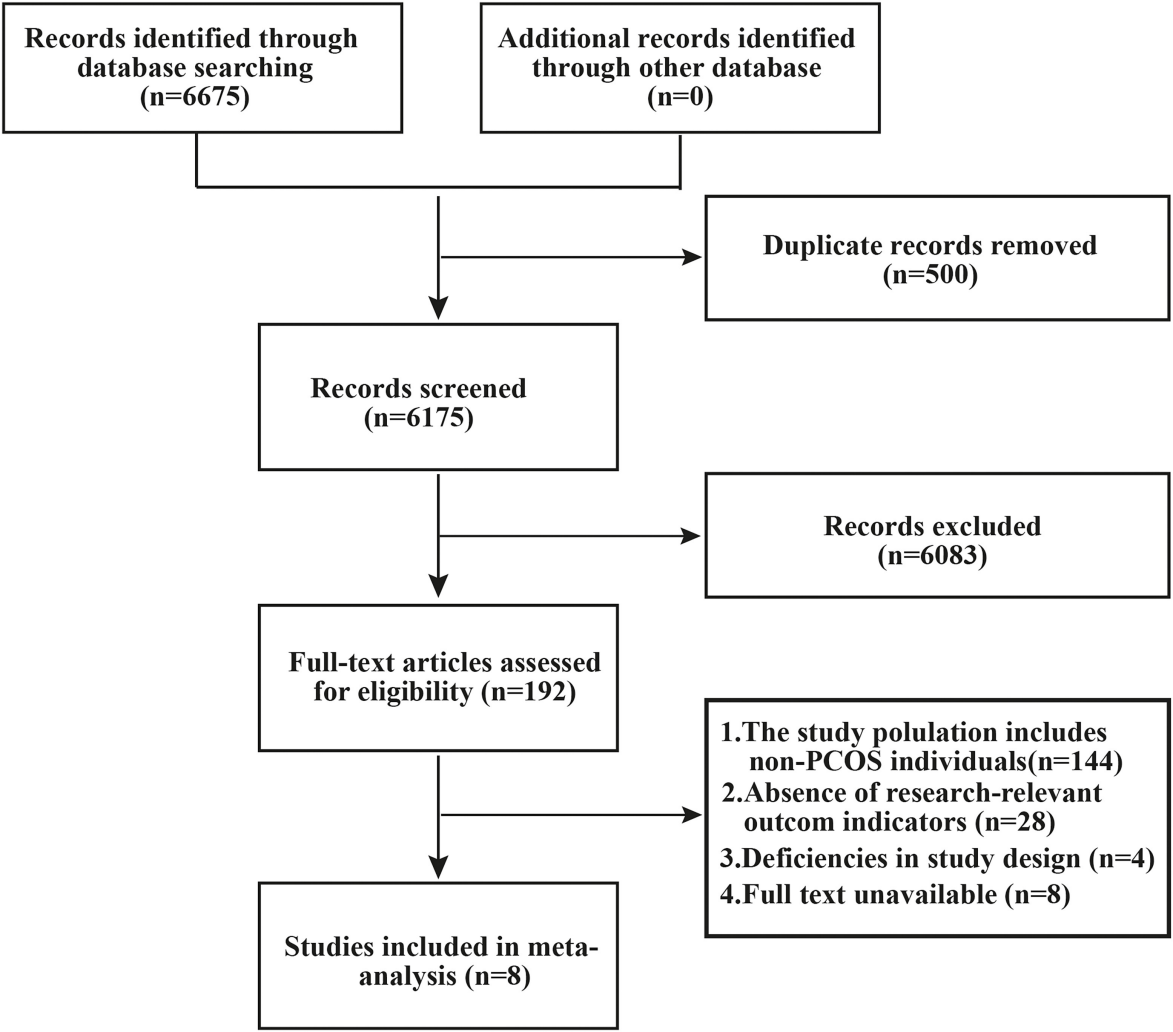
Flowchart of study selection.

From an initial yield of 6,675 records and after the removal of 500 duplicates, 6,175 unique citations were screened. Following the application of eligibility criteria that excluded non-observational studies, 192 full-text articles were assessed, of which eight fulfilled the eligibility criteria and were included (Figure 1).

The included studies, comprising a total of 6,072 women with PCOS, were conducted in several countries, including China (five studies), the United States, the United Kingdom, and Portugal (one study each). The included studies employed heterogeneous criteria for categorizing serum AMH levels. Five studies stratified patients into three groups based on percentiles: low (<25*^th^* percentile), medium (25–75*^th^* percentile), and high (>75*^th^* percentile). In contrast, three studies utilized a single cut-off value to define only low and high AMH groups. The reported outcomes varied across studies: eight studies reported the live birth rate, seven reported clinical pregnancy rates, five reported the number of oocytes retrieved, four each reported cumulative live birth rates and miscarriage rates, three studies reported the incidence of OHSS, and two reported good-quality embryo counts (Table 1).

**TABLE 1:** Characteristics of included studies in meta-analysis.

References	Country	Design	Number of cases	Outcome	AMH classification
	Britain	Retrospective cohort study	96	Trigger-day endometrial thickness (ET), live birth rate, number of retrieved oocytes, number of metaphase II (MII) oocytes, fertilization rate, number of embryos transferred.	Group1: AMH ≤ 7 ng/mL Group2: AMH ≥ 7 ng/mL
Vale-Fernandes et al. (7)	Portugal	Retrospective observational study	150	Live birth rate, cumulative live birth rate, clinical pregnancy rate	Low: AMH < 3.7 ng/ml Middle: AMH:3.7–7.4 ng/mL High: AMH > 7.4 ng/mL
Huang et al. (9)	China	Retrospective cohort study	825	Number of oocytes retrieved, clinical pregnancy, OHSS rate	Low: AMH ≤ 9.3 ng/mL High: AMH > 9.3 ng/mL
Su et al. (8)	China	Retrospective cohort study	344	Live birth rate, cumulative live birth rates, number of retrieved eggs, clinical pregnancy, early abortion, late abortion	Low: AMH: 1–12 ng/mL High: AMH: 12–25 ng/mL
Tal et al. (10)	America	Retrospective cohort study	184	Live birth rate, implantation rate, clinical pregnancy rate, number of oocytes retrieved, OHSS rate	Low: AMH ≤ 3.32 ng/mL Average: 3.32 ng/mL ≤ AMH < 8.27 ng/mL High: AMH ≥ 8.27 ng/mL
Liu et al. (35)	China	Retrospective cohort study	418	Implantation rate, clinical pregnancy rate, miscarriage rate, live birth rate	Low: AMH ≤ 4.91 ng/mL; Average: AMH: 4.91–10.88 ng/mL; High: AMH > 10.88 ng/mL
Guo et al. (22)	China	Retrospective study	2,436	Live birth rate, cumulative live birth rates, clinical pregnancy rate, normal fertilization rate, number of oocytes retrieved	Group 1: AMH ≤ 6.77 ng/ml Group 2: AMH: 6.77–14.30 ng/ml Group 3: AMH > 14.30 ng/ml
Zhao et al. (21).	China	Retrospective analysis	4,719	Live birth rate, clinical pregnancy rate, miscarriage rate, cumulative live birth rates, number of oocytes retrieved, OHSS rates	Low: AMH ≤ 4.98 ng/mL Average: AMH: 4.98–10.65 ng/mL High: AMH ≥ 10.65 ng/mL

### 3.2 Assessment of risk of bias

Each study received a score of 0–9 stars, with one star awarded per fulfilled criterion. Studies scoring ≥5 stars were considered high quality. All included articles achieved NOS scores ranging from 6 to 8, confirming high methodological quality. The results of the risk of bias for the studies included in the meta-analysis were shown in Supplementary Table 2. All the eight studies achieved a total score of 6–8, indicating generally good quality.

### 3.3 End point analysis

#### 3.3.1 Cumulative live birth rates

Meta-analysis of cumulative live birth rates (CLBR) revealed no significant differences across AMH level comparisons (Supplementary Figure 1). Substantial heterogeneity was observed for the comparison of low versus high AMH levels (I^2^ = 71%). Sensitivity analysis with sequential single-study exclusion was conducted to explore the source of heterogeneity. Removing any individual study failed to substantially reduce high between-study heterogeneity, and the source of elevated heterogeneity could not be identified. Therefore, a random-effects model was adopted for pooled analysis. The pooled risk ratio (RR) for low versus high AMH levels was 1.03 (95% CI: 0.91–1.16; Z = 0.48, *P* = 0.63), indicating no significant association with CLBR (Supplementary Figure 1A). Similarly, substantial heterogeneity was observed for the comparison of low versus medium AMH levels (I^2^ = 76%). Sensitivity analysis by sequentially omitting individual studies was performed to explore the source of heterogeneity. After excluding one study (7), no obvious heterogeneity was observed among the remaining two studies (I^2^ = 0%). However, the clinical factor responsible for the heterogeneity could not be identified, and thus a random-effects model was applied for pooled analysis. The comparison between low and medium AMH levels yielded an RR of 1.02 (95% CI: 0.89–1.17; Z = 0.30, *P* = 0.77) (Supplementary Figure 1B). In the analysis of medium versus high AMH levels, the RR was 0.98 (95% CI: 0.94–1.04; Z = 0.60, *P* = 0.55), again suggesting no significant difference in CLBR, and heterogeneity in this subgroup was low (I^2^ = 0%) (Supplementary Figure 1C).

Overall, the results suggest that AMH levels are not significantly associated with CLBR in women with PCOS. However, the considerable heterogeneity in the low-versus-high and low-versus-medium comparisons underscores potential variations in study design or patient populations, which should be considered when interpreting these findings.

#### 3.3.2 Live birth rates

**FIGURE 2:**
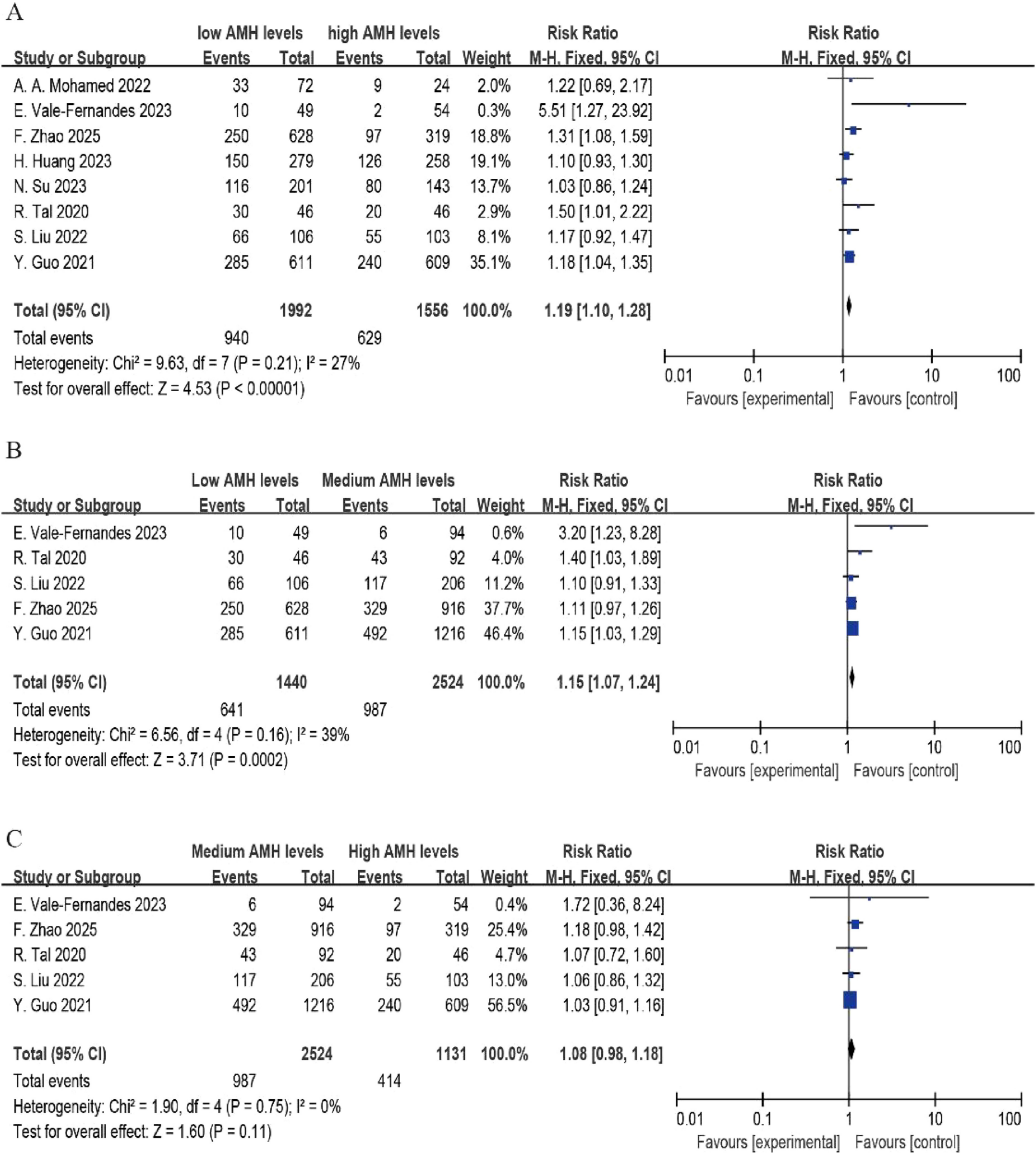
Forest plot of comparison of live birth rates among different anti-Müllerian hormone (AMH) levels in polycystic ovary syndrome (PCOS) patients. **(A)** The comparison of low versus high AMH levels. **(B)** The comparison of low versus medium AMH levels. **(C)** The comparison of medium versus high AMH levels.

Meta-analysis of live birth rates revealed a significant advantage for patients with lower AMH levels. In the comparison of low versus high AMH levels, the pooled RR was 1.19 (95% CI: 1.10–1.28; Z = 4.53, *P* < 0.00001), indicating a significantly higher live birth rate in the low AMH group, with moderate heterogeneity (I^2^ = 27%) (Figure 2A). Similarly, low AMH was associated with a higher live birth rate compared to medium AMH levels (RR = 1.15, 95% CI: 1.07–1.24; Z = 3.71, *P* = 0.0002), also with moderate heterogeneity (I^2^ = 39%) (Figure 2B). In contrast, the difference between medium and high AMH levels was not statistically significant (RR = 1.08, 95% CI: 0.98–1.18; Z = 1.60, *P* = 0.11), and heterogeneity was low (I^2^ = 0%) (Figure 2C).

In summary, these results suggest that in women with PCOS, low AMH levels are associated with significantly higher live birth rates relative to both medium and high AMH levels, whereas no significant difference was observed between medium and high AMH groups. These findings may inform clinical counseling and management strategies for PCOS patients undergoing fertility treatment. Targeted subgroup analysis limited to low versus high AMH comparison among the five studies adopting consistent percentile grouping criteria (low < 25%, medium 25%–75%, high > 75%) generated findings consistent with the overall pooled results (Supplementary Figure 2), further verifying the reliability of our primary results.

#### 3.3.3 Clinical pregnancy rate

**FIGURE 3:**
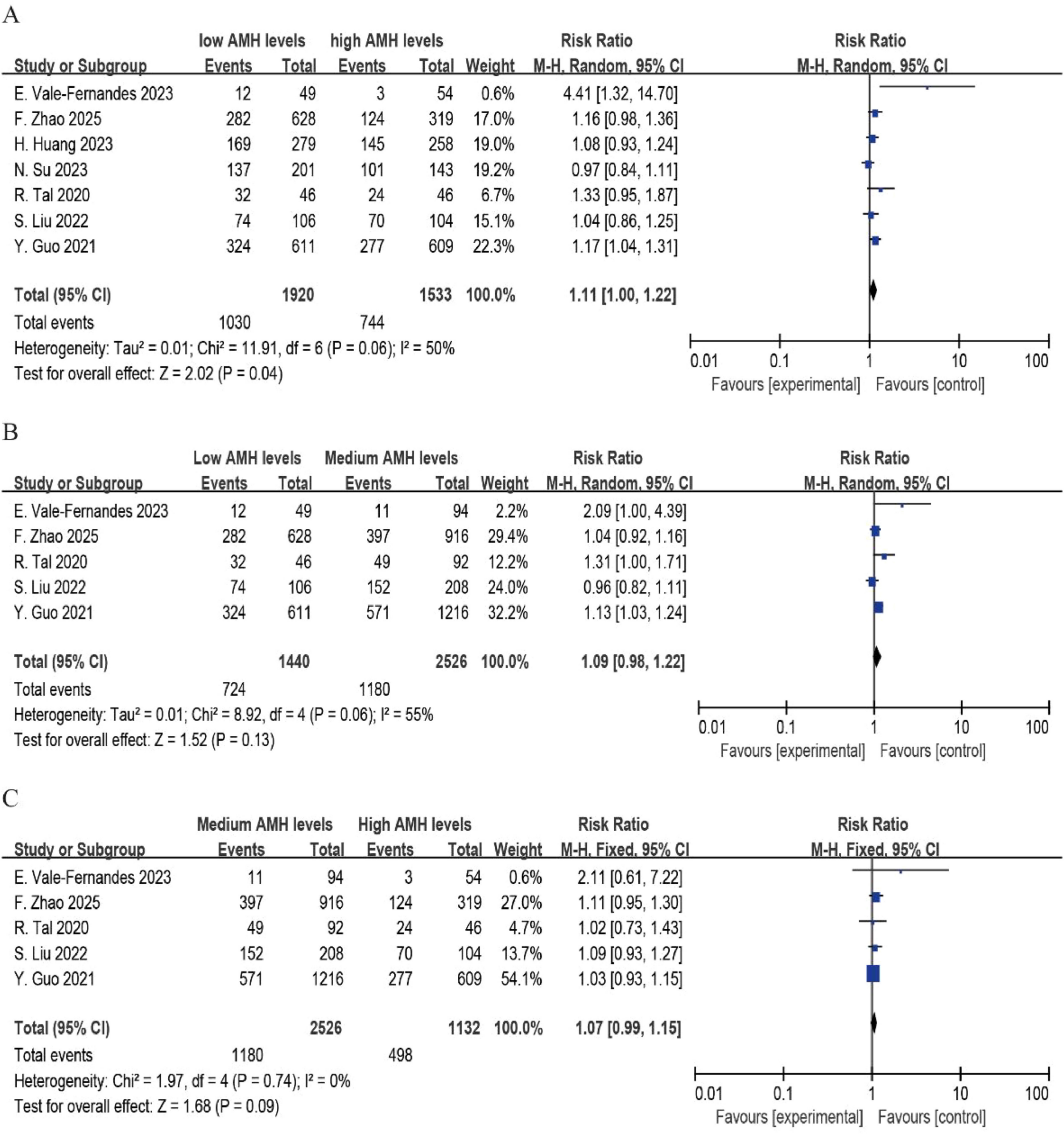
Forest plot of pooled risk ratios for clinical pregnancy rates across anti-Müllerian hormone (AMH) levels in polycystic ovary syndrome (PCOS) patients. **(A)** The comparison of low versus high AMH levels. **(B)** The comparison of low versus medium AMH levels. **(C)** The comparison of medium versus high AMH levels.

Overall, meta-analysis revealed only a weak association between AMH levels and clinical pregnancy rates in PCOS patients. The sole comparison showing a trend was between low and high AMH levels, yielding a pooled RR of 1.11 (95% CI: 1.00–1.22; *P* = 0.04), with moderate heterogeneity (I^2^ = 50%) (Figure 3A). No significant differences were found for low versus medium AMH (RR = 1.09, *P* = 0.13) or medium versus high AMH (RR = 1.07, *P* = 0.09) (Figures 3B,C). Removing any individual study failed to substantially reduce high between-study heterogeneity, and the source of elevated heterogeneity could not be identified.

These results suggest that any potential link between lower AMH and higher clinical pregnancy rates is not robust across comparisons, highlighting the need for cautious interpretation in clinical counseling.

#### 3.3.4 Miscarriage rate

**FIGURE 4:**
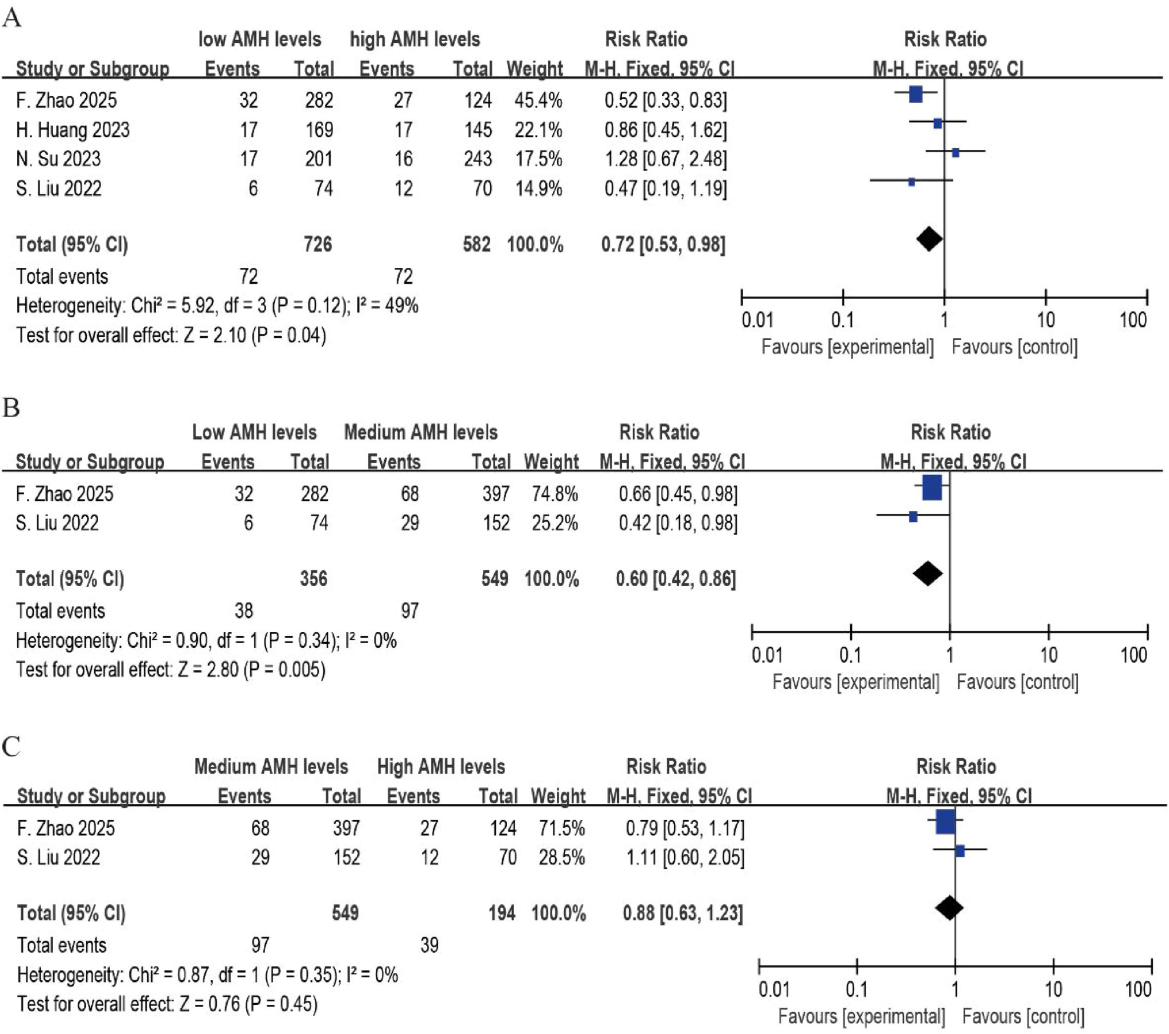
Forest plot for the comparison of miscarriage risk among different anti-Müllerian hormone (AMH) levels in polycystic ovary syndrome (PCOS) patients. **(A)** The comparison of low versus high AMH levels. **(B)** The comparison of low versus medium AMH levels. **(C)** The comparison of medium versus high AMH levels.

Meta-analysis indicated that lower AMH levels were significantly associated with a lower miscarriage risk among women with PCOS. The pooled risk ratio (RR) for miscarriage was significantly lower in the low versus high AMH group (RR = 0.72, 95% CI: 0.53–0.98; *Z* = 2.10, *P* = 0.04), with moderate heterogeneity (I^2^ = 49%) (Figure 4A). A more pronounced reduction was observed in the comparison of low versus medium AMH levels (RR = 0.60, 95% CI: 0.42–0.86; *Z* = 2.80, *P* = 0.005), with no heterogeneity (I^2^ = 0%) (Figure 4B). In contrast, no significant difference was found between medium and high AMH levels (RR = 0.88, 95% CI: 0.63–1.23; *Z* = 0.76, *P* = 0.45), also with no heterogeneity (I^2^ = 0%) (Figure 4C).

In summary, these results indicate that low AMH levels are associated with a significantly lower risk of miscarriage compared to both medium and high AMH levels in women with PCOS, whereas the miscarriage risk did not differ significantly between medium and high AMH groups. These findings enhance our understanding of the prognostic role of AMH in early pregnancy outcomes among PCOS patients.

#### 3.3.5 Number of oocytes retrieved

Meta-analysis showed that oocyte yield tended to increase across sequentially elevated AMH subgroups among patients with PCOS, with statistically significant between-group differences. The pooled mean difference in the number of oocytes retrieved was −3.91 (95% CI: −5.60 to −2.23; *Z* = 4.55, *P* < 0.001) for low versus high AMH levels (Supplementary Figure 3A), indicating a substantially higher oocyte yield in the high AMH group. Similarly, patients with medium AMH levels retrieved significantly more oocytes than those with low AMH levels (MD = −2.33, 95% CI: −2.79 to −1.86; *Z* = 9.77, *P* < 0.001; Supplementary Figure 3B). A significant difference was also observed between medium and high AMH levels (MD = −1.70, 95% CI: −3.31 to −0.09; *Z* = 2.07, *P* = 0.04; Supplementary Figure 3C), confirming that oocyte retrieval numbers increase with ascending AMH categories. These results indicate a strong positive association between AMH levels and oocyte retrieval, supporting its role as a key predictor of ovarian response in PCOS patients undergoing controlled ovarian stimulation.

We further examined the association between varying levels of AMH and the number of good-quality embryos in women with PCOS. Given the limited availability of relevant studies and the paucity of data specifically addressing medium AMH levels, our analysis focused exclusively on comparing low versus high AMH levels. The pooled mean difference in the count of good-quality embryos between high and low AMH groups was −0.98 (95% CI: −2.94 to 0.98; *Z* = 0.98, *P* = 0.33; Supplementary Figure 3D), indicating no statistically significant difference in the number of good-quality embryos between PCOS patients with low and high AMH levels. Notably, substantial heterogeneity was observed among the included studies (I^2^ = 95%), suggesting considerable variability in the reported outcomes.

#### 3.3.6 Incidence of OHSS

Meta-analysis of OHSS incidence revealed significant associations with AMH levels in PCOS patients. In this part of analysis, the former group in each comparison was set as the reference group. The pooled OR of low AMH (reference) versus high AMH was 0.64 (95% CI: 0.47–0.88; Z = 2.77, *P* = 0.006). Since OR < 1, it demonstrated that the high AMH group had a significantly higher OHSS risk than the low AMH group (Supplementary Figure 4A). For medium AMH (reference) versus high AMH, the pooled OR was 0.40 (95% CI: 0.23–0.70; Z = 3.19, *P* = 0.001), which also indicated an elevated OHSS risk in patients with high AMH (Supplementary Figure 4C). No significant difference in OHSS risk was observed between low and medium AMH levels (OR = 1.29, 95% CI: 0.67–2.49; Z = 0.76, *P* = 0.45) (Supplementary Figure 4B).

Overall, these results suggest that in women with PCOS, higher AMH levels are associated with a significantly increased risk of developing OHSS compared to both low and medium AMH levels, whereas the risk does not differ significantly between low and medium AMH groups.

## 4 Discussion

As a marker of ovarian reserve, AMH is thought to regulates folliculogenesis by inhibiting the recruitment of primary follicles and selection of small antral follicles (11). Patients with PCOS have an increased number of preantral and small antral follicles, which results in intrafollicular and serum AMH levels that are 2–4 times higher than those in women without PCOS (12). Given the positive correlation between AMH and antral follicle count, the 2023 international evidence-based PCOS guideline endorses AMH as a diagnostic tool that can be used as an alternative to ultrasound (13). However, AMH levels are influenced by numerous factors, which contributes to their significant heterogeneity across different populations (14). The rationale for focusing on the relationship between AMH levels and pregnancy outcomes in PCOS lies in the established role of AMH as a biomarker of PCOS severity. This is evidenced by its positive correlation with symptom severity and its significant elevation in the context of hyperandrogenism (15, 16). Furthermore, studies have reported a positive correlation of AMH levels with both the LH/FSH ratio (17) and testosterone levels (18) in PCOS.

Anti-Müllerian hormone has been proposed as a predictive indicator of ovarian response and the number of oocytes retrieved to ovarian hormonal stimulation (19, 20). Our results indicate a strong positive association between AMH levels and oocyte retrieval in patients with PCOS. The association between AMH levels and oocyte yield was most pronounced when comparing PCOS patients with high versus low AMH levels, as well as those with low versus medium AMH levels (8, 9, 21, 22). However, the difference in oocyte retrieval between medium and high AMH groups remains inconsistent across studies. For instance, Zhao et al. (21). reported a significantly higher oocyte number in patients with medium AMH levels compared to those with low AMH levels, whereas studies by Tal et al. (10), Guo et al. (22) found no significant difference between these two groups. These discrepant findings may reflect variations in sample size, patient characteristics, or AMH stratification criteria, underscoring the need for standardized protocols in future investigations. Furthermore, excessively elevated AMH levels exacerbate hyperandrogenism, thereby collectively accelerating the arrest of follicular development. Mechanistically, studies indicate that a hyperandrogen-induced imbalance in the FOXO4-AR regulatory loop contributes significantly to the ovulatory dysfunction observed in PCOS (23). This pathophysiological mechanism has been proposed in the literature to explain why quantitative advantages in follicle count may not always translate into improved outcomes, but this remains speculative in the context of the present data.

Embryo developmental potential is a crucial determinant of implantation and ongoing pregnancy. However, AMH is generally considered to have limited predictive value for embryo quality. Our meta-analysis did not identify a significant association between AMH levels and the number of good-quality embryos in PCOS patients. Zhao reported the higher (>10.65 ng/ml) and medium (4.98–10.65 ng/ml) AMH contributes a greater number of good-quality embryos in women with PCOS (21). A study conducted on the general population revealed that individuals with high AMH levels (>5.6 ng/ml) had a significantly higher number of mature follicles compared to those with moderate (1.61–5.6 ng/ml) or low AMH level (≤1.61 ng/ml) (24). While the greater number of mature follicles does not directly translate to superior embryo quality. Consistent with this, studies have demonstrated that age, rather than AMH, is the primary influencing factor on embryo quality (25). This pattern is also observed among young patients with diminished ovarian reserve (DOR): low AMH (<1.2 ng/ml) is associated with fewer retrieved oocytes, while no evident association exists between AMH and oocyte developmental competence (26). Furthermore, evidence confirms that AMH levels shows no association with blastocyst aneuploidy rates, underscoring its inability to predict the chromosomal integrity of embryos (27).

Anti-Müllerian hormone has been widely recognized as a predictor of OHSS in controlled ovarian stimulation and assisted reproductive technology (28). Previous study revealed that the cancellation rate due to OHSS risk was significantly higher in patients with AMH > 12 ng/ml than those with AMH < 12 ng/ml, while in PCOS patients, AMH was not associated with higher odds of transfer cancellation because of OHSS (29). Our updated conclusion that high AMH increases OHSS risk is consistent with mainstream clinical experience and previous literature focused on PCOS populations. As a well-recognized biomarker of ovarian follicle reserve, AMH is positively correlated with the number of antral follicles. PCOS patients with excessively high AMH usually have excessive follicle recruitment after controlled ovarian stimulation, which greatly raises the susceptibility to OHSS. A number of prior studies have verified that AMH is a powerful predictive indicator for OHSS: patients with AMH above 12 ng/mL presented a remarkably higher OHSS risk and higher cycle cancellation rate due to OHSS, which is in line with our pooled results (30). In the present meta-analysis, low and medium AMH groups showed similar OHSS risk, which may be attributed to the relatively mild follicle hyperplasia and stable ovarian response in these two subgroups. Collectively, our findings reconfirm that high AMH is an important clinical warning marker for OHSS in PCOS patients undergoing ART, and these findings may help clinicians in considering individualized ovarian stimulation protocols for patients with elevated AMH to reduce OHSS complications.

Anti-Müllerian hormone has been proposed as an independent predictor for LBR following ART (31, 32). LBR are significantly lower in women with PCOS than in non-PCOS populations (33), a difference frequently attributed to endocrine dysfunction. This is further elucidated by the role of AMH: a previous meta-analysis indicated that lower AMH levels are associated with significantly higher LBR compared to high AMH levels (30). Specifically, in PCOS population, AMH levels below 12 ng/mL show a negative correlation with both LBR and clinical pregnancy rate, a correlation that weakens when AMH exceeds this threshold. A similar pattern is observed in the general population, where an AMH level greater than 5 ng/ml is associated with a 3% decrease in LBR per unit increase (29). Reinforcing that excessively high AMH may be suboptimal for reproductive success beyond its role in predicting oocyte yield.

Women with PCOS have been reported to face 49%–53% higher odds of miscarriage compared to those without PCOS (34). While advanced maternal age and diminished ovarian reserve are generally considered risk factors for miscarriage due to declining oocyte quality, studies indicate that young women with PCOS still exhibit a significantly elevated risk of pregnancy loss compared to their non-PCOS counterparts (34). In the present meta-analysis, which focused specifically on women with PCOS, the risk of miscarriage was 39% higher in those with high AMH levels than in those with low AMH levels. This finding is consistent with previous reports by Liu et al. (35), Zhao et al. (21). However, some studies have shown no association between miscarriage rates and AMH levels in patients with PCOS (8, 9). Underlying mechanisms may involve chronic inflammation and metabolic disturbances, which are associated with impaired endometrial decidualization and diminished endometrial receptivity, conditions that have been linked to elevated miscarriage risk in patients with PCOS (36).

The complex relationship between AMH and IVF outcomes in PCOS arises from the fundamental endocrine and metabolic disturbances that characterize the disorder, including insulin resistance (IR), dyslipidemia, and hyperandrogenism. These imbalances concurrently impair follicular development and disrupt endometrial receptivity, largely through defective stromal decidualization. Consequently, they contribute to a range of adverse reproductive outcomes, such as implantation failure, miscarriage, preterm birth, and an increased risk of gestational complications like preeclampsia (37). Endometrial dysregulation in PCOS is further compounded by dysregulation of steroid hormone receptors. Specifically, elevated ER-α expression promotes hyperproliferation (38), while simultaneous upregulation of progesterone receptor (PR-α, PR-β) and androgen receptor (AR) (39) underlies progesterone resistance and delayed decidualization (40). Insulin resistance in endometrium represents another key mechanism impairing decidualization. Studies indicate that IR downregulates GLUT-4, a glucose transporter critical for cellular glucose uptake, thereby disrupting glucose metabolism and reducing endometrial receptivity (41). This metabolic dysfunction is exacerbated by a low-grade inflammatory state, characterized by elevated level of TNF-α, IL-6, and activation of NF-κB. In PCOS patients, increased TNF-α suppresses endometrial GLUT-4 expression via inthe activation of p-NF-κBp65 signaling pathway, further delaying the embryo implantation (42). Furthermore, mitochondrial dysfunction is strongly implicated in the pathophysiology of PCOS. By disrupting energy metabolism and exacerbating oxidative stress, it inflicts damage on endometrial cells, thereby impairing their receptivity and functional capacity (43).

One major limitation of the present meta-analysis is inconsistent AMH grouping standards across included studies. Five studies stratified patients by population AMH percentile, while another three adopted distinct fixed AMH cut-off values for binary grouping with thresholds varying from 3.32 to 14.30 ng/mL, which may affect result comparability and partially contribute to inter-study heterogeneity. To explore the influence of percentile grouping on primary outcomes, we conducted a targeted subgroup analysis limited to live birth rate, only comparing low versus high AMH groups among the five percentile-based studies; subgroup analyses for other endpoints and other pairwise comparisons were not performed. The pooled effect size of this percentile subgroup was consistent with the overall meta-analysis results of all included studies, confirming the stability of our main findings. Due to inconsistent cut-off values and limited sample size, subgroup analysis was abandoned for three fixed-cutoff studies. Uniform AMH stratification criteria are needed in future PCOS-related researches to reduce grouping heterogeneity. Second, various confounders including BMI, insulin resistance, ovarian stimulation regimens and metabolic characteristics may interfere with reproductive outcomes and act as critical confounding sources in our pooled analysis. Third, all enrolled studies were observational, so we can only identify statistical associations instead of causal links between AMH and ART outcomes; further prospective randomized trials are required to verify potential causality. Lastly, analyses of OHSS, miscarriage and embryo quality were restricted by limited study quantity alongside moderate-to-high heterogeneity, and relevant subgroup results should be interpreted as exploratory rather than conclusive.

## 5 Conclusion

This meta-analysis reveals a complex and paradoxical pattern in the association between AMH levels and ART outcomes in PCOS patients. On one hand, AMH robustly predicts quantitative aspects of ovarian stimulation, demonstrating a strong stepwise positive association with oocyte yield; nevertheless, high AMH levels correspond to increased susceptibility to OHSS in PCOS patients. On the other hand, its relationship with qualitative reproductive outcomes is inverse; notably, low AMH levels are associated with significantly higher live birth rates and a lower risk of miscarriage compared to both medium and high AMH levels, with the medium AMH group consistently exhibiting an intermediate prognosis. No significant association was observed with cumulative live birth or clinical pregnancy rates. These findings collectively suggest that within the PCOS population, a higher AMH level, while suggestive of a stronger ovarian response and greater oocyte acquisition, is a biomarker linked to poorer reproductive and safety outcomes, which suggests that refined clinical interpretation may be warranted, moving beyond its use solely for predicting ovarian reserve.

## Data Availability

The original contributions presented in this study are included in this article/Supplementary material, further inquiries can be directed to the corresponding author.

## Supplementary material

Supplementary Figure 1 Forest plot of pooled risk ratios for cumulative live birth rates across anti-Müllerian hormone (AMH) levels in polycystic ovary syndrome (PCOS) patients.

Supplementary Figure 2 Subgroup analysis of live birth rate [low vs. high anti-Müllerian hormone (AMH)] restricted to five percentile-stratified studies in polycystic ovary syndrome (PCOS) patients.

Supplementary Figure 3 Comparison of the number of oocytes retrieved among polycystic ovary syndrome (PCOS) patients with different anti-Müllerian hormone (AMH) levels.

Supplementary Figure 4 Forest plot for the association between anti-Müllerian hormone (AMH) levels and the incidence of ovarian hyperstimulation syndrome (OHSS) in polycystic ovary syndrome (PCOS) patients.

## References

[1] Pereira-Eshraghi CF Vuguin PP. Polycystic ovary syndrome. *Pediatr Rev*. (2024) 45:363–5. 10.1542/pir.2023-006036 38821890

[2] Chiaffarino F Cipriani S Dalmartello M Ricci E Esposito G Fedele F et al. Prevalence of polycystic ovary syndrome in European countries and USA: a systematic review and meta-analysis. *Eur J Obstet Gynecol Reprod Biol*. (2022) 279:159–70. 10.1016/j.ejogrb.2022.10.020 36343588

[3] Yang R Li Q Zhou Z Qian W Zhang J Wu Z et al. Changes in the prevalence of polycystic ovary syndrome in China over the past decade. *Lancet Reg Health West Pac*. (2022) 25:100494. 10.1016/j.lanwpc.2022.100494 35669932 PMC9162959

[4] Bansal S Goyal M Sharma C Shekhar S. Letrozole versus clomiphene citrate for ovulation induction in anovulatory women with polycystic ovarian syndrome: a randomized controlled trial. *Int J Gynaecol Obstet*. (2021) 152:345–50. 10.1002/ijgo.13375 32920843

[5] Sakar MN Oðlak SC. Comparison of the efficacy of letrozole stair-step protocol with clomiphene citrate stair-step protocol in the management of clomiphene citrate-resistant polycystic ovary syndrome patients. *J Obstet Gynaecol Res*. (2021) 47:3875–82. 10.1111/jog.14936 34254401

[6] Pan ML Chen LR Chen KH. The risk of subsequent miscarriage in pregnant women with prior polycystic ovarian syndrome: a nationwide population-based study. *Int J Environ Res Public Health*. (2021) 18:8253. 10.3390/ijerph18168253 34444016 PMC8394863

[7] Vale-Fernandes E Barreiro M Leal C Macedo RZ Tomé A Monteiro MP. Elevated anti-Müllerian hormone as a prognostic factor for poor outcomes of in vitro fertilization in women with polycystic ovary syndrome. *Biomedicines*. (2023) 11:3150. 10.3390/biomedicines11123150 38137371 PMC10740605

[8] Su N Zhan J Xie M Zhao Y Huang C Wang S et al. High anti-Mullerian hormone level is adversely associated with cumulative live birth rates of two embryo transfers after the first initiated cycle in patients with polycystic ovary syndrome. *Front Endocrinol (Lausanne)*. (2023) 14:1123125. 10.3389/fendo.2023.1123125 37388214 PMC10305806

[9] Huang H Gao H Shi Y Deng B He X Lin J et al. Can AMH levels predict the need to step up FSH dose for controlled ovarian stimulation following a long GnRH agonist protocol in PCOS women? *Reprod Biol Endocrinol*. (2023) 21:121. 10.1186/s12958-023-01173-8 38110998 PMC10726541

[10] Tal R Seifer CM Khanimov M Seifer DB Tal O. High serum Antimullerian hormone levels are associated with lower live birth rates in women with polycystic ovarian syndrome undergoing assisted reproductive technology. *Reprod Biol Endocrinol*. (2020) 18:20. 10.1186/s12958-020-00581-4 32156287 PMC7065318

[11] Bedenk J Vrtaènik-Bokal E Virant-Klun I. The role of anti-Müllerian hormone (AMH) in ovarian disease and infertility. *J Assist Reprod Genet*. (2020) 37:89–100. 10.1007/s10815-019-01622-7 31755000 PMC7000586

[12] Laven JS Mulders AG Visser JA Themmen AP De Jong FH Fauser BC. Anti-Müllerian hormone serum concentrations in normoovulatory and anovulatory women of reproductive age. *J Clin Endocrinol Metab*. (2004) 89:318–23. 10.1210/jc.2003-030932 14715867

[13] Teede HJ Tay CT Laven JJE Dokras A Moran LJ Piltonen TT et al. Recommendations from the 2023 international evidence-based guideline for the assessment and management of polycystic ovary syndrome. *J Clin Endocrinol Metab*. (2023) 108:2447–69. 10.1210/clinem/dgad463 37580314 PMC10505534

[14] van der Ham K Laven JSE Tay CT Mousa A Teede H Louwers YV. Anti-Müllerian hormone as a diagnostic biomarker for polycystic ovary syndrome and polycystic ovarian morphology: a systematic review and meta-analysis. *Fertil Steril*. (2024) 122:727–39. 10.1016/j.fertnstert.2024.05.163 38944177

[15] Dewailly D Pigny P Soudan B Catteau-Jonard S Decanter C Poncelet E et al. Reconciling the definitions of polycystic ovary syndrome: the ovarian follicle number and serum anti-Müllerian hormone concentrations aggregate with the markers of hyperandrogenism. *J Clin Endocrinol Metab*. (2010) 95:4399–405. 10.1210/jc.2010-0334 20610596

[16] Rudnicka E Kunicki M Calik-Ksepka A Suchta K Duszewska A Smolarczyk K et al. Anti-Müllerian hormone in pathogenesis, diagnostic and treatment of PCOS. *Int J Mol Sci*. (2021) 22:12507. 10.3390/ijms222212507 34830389 PMC8619458

[17] Pratama G Wiweko B Asmarinah, Widyahening IS Andraini T Bayuaji H et al. Mechanism of elevated LH/FSH ratio in lean PCOS revisited: a path analysis. *Sci Rep*. (2024) 14:8229. 10.1038/s41598-024-58064-0 38589425 PMC11002031

[18] Li Y Zhai Y Li L Lu Y Su S Liu Y et al. Divergent associations between serum androgens and ovarian reserve markers revealed in patients with polycystic ovary syndrome. *Front Endocrinol (Lausanne)*. (2022) 13:881740. 10.3389/fendo.2022.881740 35757414 PMC9218193

[19] Arce JC Andersen AN Fernández-Sánchez M Visnova H Bosch E García-Velasco JA et al. Ovarian response to recombinant human follicle-stimulating hormone: a randomized, antimüllerian hormone-stratified, dose-response trial in women undergoing in vitro fertilization/intracytoplasmic sperm injection. *Fertil Steril*. (2014) 102:1633–40.e5. 10.1016/j.fertnstert.2014.08.013 25256937

[20] Sun XY Lan YZ Liu S Long XP Mao XG Liu L. Relationship between anti-Müllerian hormone and in vitro fertilization-embryo transfer in clinical pregnancy. *Front Endocrinol (Lausanne)*. (2020) 11:595448. 10.3389/fendo.2020.595448 33343511 PMC7746804

[21] Zhao F Wen D Zeng L Wang R Wang D Xu H et al. High anti-Müllerian hormone level as a predictor of poor pregnancy outcomes in women with polycystic ovary syndrome undergoing in vitro fertilization/intracytoplasmic sperm injection: a retrospective cohort study. *Reprod Biol Endocrinol*. (2025) 23:15. 10.1186/s12958-025-01347-6 39875902 PMC11773973

[22] Guo Y Liu S Hu S Li F Jin L. High serum anti-Müllerian hormone concentrations are associated with poor pregnancy outcome in fresh IVF/ICSI cycle but not cumulative live birth rate in PCOS patients. *Front Endocrinol (Lausanne)*. (2021) 12:673284. 10.3389/fendo.2021.673284 34122349 PMC8187895

[23] Zhou R Wang Z Dong M Wang S Li X Huang J et al. Hyperandrogen-induced imbalance of FOXO4-AR regulatory loop contributes to ovulatory disorders in polycystic ovary syndrome. *Mol Ther Nucleic Acids*. (2025) 36:102543. 10.1016/j.omtn.2025.102543 40520361 PMC12166420

[24] Karacin P Dilbaz S Aldemir O Dilbaz B Ustun YE. Is there a relationship between serum anti-Mullerian hormone levels and abortion rates in patients who received in vitro fertilisation-embryo transfer cycles? *J Hum Reprod Sci*. (2023) 16:57–63. 10.4103/jhrs.jhrs_17_23 37305769 PMC10256938

[25] Dai X Wang Y Yang H Gao T Yu C Cao F et al. AMH has no role in predicting oocyte quality in women with advanced age undergoing IVF/ICSI cycles. *Sci Rep*. (2020) 10:19750. 10.1038/s41598-020-76543-y 33184364 PMC7661530

[26] Wang MJ Lin MH Yang JH Lee RK Lee KS. Low antimüllerian hormone (<1.2 ng/ml) does not impact oocyte quality and IVF/ICSI outcomes in women =40 years old. *Taiwan J Obstet Gynecol*. (2025) 64:248–52. 10.1016/j.tjog.2024.02.007 40049808

[27] Pipari A Guillen A Cruz M Pacheco A Garcia-Velasco JA. Serum anti-Müllerian hormone levels are not associated with aneuploidy rates in human blastocysts. *Reprod Biomed Online*. (2021) 42:1211–8. 10.1016/j.rbmo.2021.03.006 33849787

[28] Fu H Lin Y Deng X Wu L. Correlation between anti-Mullerian hormone levels and antral follicle counts in polycystic ovary and metabolic syndromes. *Syst Biol Reprod Med*. (2021) 67:112–20. 10.1080/19396368.2020.1860155 33406916

[29] Acharya KS Harris BS Weber JM Truong T Pieper C Eaton JL. Impact of increasing antimüllerian hormone level on in vitro fertilization fresh transfer and live birth rate. *F S Rep*. (2022) 3:223–30. 10.1016/j.xfre.2022.06.005 36212572 PMC9532892

[30] Yuwen T Yang Z Cai G Feng G Liu Q Fu H. Association between serum AMH levels and IVF/ICSI outcomes in patients with polycystic ovary syndrome: a systematic review and meta-analysis. *Reprod Biol Endocrinol*. (2023) 21:95. 10.1186/s12958-023-01153-y 37872575 PMC10591359

[31] Tal R Seifer DB Wantman E Baker V Tal O. Antimüllerian hormone as a predictor of live birth following assisted reproduction: an analysis of 85,062 fresh and thawed cycles from the Society for Assisted Reproductive Technology Clinic Outcome Reporting System database for 2012-2013. *Fertil Steril*. (2018) 109:258–65. 10.1016/j.fertnstert.2017.10.021 29331235

[32] Peigné M Bernard V Dijols L Creux H Robin G Hocké C et al. Using serum anti-Müllerian hormone levels to predict the chance of live birth after spontaneous or assisted conception: a systematic review and meta-analysis. *Hum Reprod*. (2023) 38:1789–806. 10.1093/humrep/dead147 37475164

[33] Li T An H Yao G Dai W Zhang R Chu T et al. Live birth rates in IVF patients with and without polycystic ovary syndrome according to serum anti-Müllerian hormone concentrations. *Reprod Biomed Online*. (2025) 50:104731. 10.1016/j.rbmo.2024.104731 40350354

[34] Bahri Khomami M Shorakae S Hashemi S Harrison CL Piltonen TT Romualdi D et al. Systematic review and meta-analysis of pregnancy outcomes in women with polycystic ovary syndrome. *Nat Commun*. (2024) 15:5591. 10.1038/s41467-024-49749-1 38965226 PMC11224312

[35] Liu S Hong L Mo M Xiao S Wang X Fan X et al. Association of antimüllerian hormone with polycystic ovarian syndrome phenotypes and pregnancy outcomes of in vitro fertilization cycles with fresh embryo transfer. *BMC Pregnancy Childbirth*. (2022) 22:171. 10.1186/s12884-022-04518-0 35236324 PMC8892693

[36] Zhao J Chen Q Xue X. An update on the progress of endometrial receptivity in women with polycystic ovary syndrome. *Reprod Sci*. (2022) 29:2136–44. 10.1007/s43032-021-00641-z 34076874

[37] Palomba S Piltonen TT Giudice LC. Endometrial function in women with polycystic ovary syndrome: a comprehensive review. *Hum Reprod Update*. (2021) 27:584–618. 10.1093/humupd/dmaa051 33302299

[38] Xu XL Deng SL Lian ZX Yu K. Estrogen receptors in polycystic ovary syndrome. *Cells*. (2021) 10:459. 10.3390/cells10020459 33669960 PMC7924872

[39] Wang K Li Y Chen Y. Androgen excess: a hallmark of polycystic ovary syndrome. *Front Endocrinol (Lausanne)*. (2023) 14:1273542. 10.3389/fendo.2023.1273542 38152131 PMC10751361

[40] Younas K Quintela M Thomas S Garcia-Parra J Blake L Whiteland H et al. Delayed endometrial decidualisation in polycystic ovary syndrome; the role of AR-MAGEA11. *J Mol Med (Berl)*. (2019) 97:1315–27. 10.1007/s00109-019-01809-6 31256208 PMC6713698

[41] Li J Chen S Qin R Liu X Fan L Wei M et al. Talin1 regulates glucose metabolism and endometrial receptivity via GLUT-4 in patients with polycystic ovary syndrome and insulin resistance. *Gynecol Endocrinol*. (2023) 39:2231085. 10.1080/09513590.2023.2231085 37395213

[42] LX Wu YY Yin T Yuan YY Du YD. Effect of TNF-alpha on endometrial glucose transporter-4 expression in patients with polycystic ovary syndrome through nuclear factor-kappa B signaling pathway activation. *J Physiol Pharmacol.* (2021) 72:965–73. 10.26402/jpp.2021.6.13 35485360

[43] Dabravolski SA Nikiforov NG Eid AH Nedosugova LV Starodubova AV Popkova TV et al. Mitochondrial dysfunction and chronic inflammation in polycystic ovary syndrome. *Int J Mol Sci*. (2021) 22:3923. 10.3390/ijms22083923 33920227 PMC8070512

