# Supplementary material for: Anti-Müllerian hormone levels and live birth rate in polycystic ovary syndrome patients undergoing assisted reproductive technology: a systematic review and meta-analysis
Source: Front Med (Lausanne). 2026 Jul 28;13:1859573. doi: 10.3389/fmed.2026.1859573 (PMC13457698; doi:10.3389/fmed.2026.1859573)
Supplement: Supplementary file 1 [file Table_1.docx]

**Supplementary Material**

Supplementary table 1. Search strategy

| **Database** | **Query** | **Results** |
| --- | --- | --- |
| **Embase** |  |  |
| #1 | ('polycystic ovary syndrome'/exp OR 'pcos':ti,ab,kw OR 'stein-leventhal syndrome':ti,ab,kw) AND ('anti mullerian hormone'/exp OR 'amh':ti,ab,kw OR 'müllerian inhibiting substance':ti,ab,kw) AND ('in vitro fertilization'/exp OR 'ivf':ti,ab,kw OR 'intracytoplasmic sperm injection'/exp OR 'icsi':ti,ab,kw) AND ('pregnancy outcome'/exp OR 'clinical pregnancy rate':ti,ab,kw OR 'live birth':ti,ab,kw OR 'miscarriage'/exp OR 'spontaneous abortion':ti,ab,kw) AND ('cohort analysis'/exp OR 'case control study'/exp OR 'observational study':ti,ab,kw OR 'prospective study':ti,ab,kw) | 208 |
| **Cochrane** |  |  |
| #1 | "anti-mullerian hormone"[Mesh] OR "AMH"[tiab] OR "anti mullerian hormone"[tiab] OR "müllerian-inhibiting substance"[tiab] | 51 |
| **Web of Science** |  |  |
| #1 | "anti-mullerian hormone"[Mesh] OR "AMH"[tiab] OR "anti mullerian hormone"[tiab] OR "müllerian-inhibiting substance"[tiab] | 71 |
| **PubMed** |  |  |
| #1 | "anti-mullerian hormone"[Mesh] OR "AMH"[tiab] OR "anti mullerian hormone"[tiab] OR "müllerian-inhibiting substance"[tiab] | 3,778 |
| #2 | "polycystic ovary syndrome"[Mesh] OR "polycystic ovarian syndrome"[tiab] OR "PCOS"[tiab] OR "Rotterdam criteria"[tiab] | 8,105 |
| #3 | "fertilization in vitro"[Mesh] OR "IVF"[tiab] OR "sperm injections, intracytoplasmic"[Mesh] OR "ICSI"[tiab] OR "assisted reproductive technology"[tiab] | 14,281 |
| #4 | "pregnancy outcome"[Mesh] OR "pregnancy rate"[tiab] OR "live birth"[tiab] OR "clinical pregnancy"[tiab] OR "miscarriage"[tiab] OR "abortion, spontaneous"[Mesh] | 27,287 |
| #5 | （"anti-mullerian hormone"[Mesh] OR "AMH"[tiab] OR "anti mullerian hormone"[tiab] OR "müllerian-inhibiting substance"[tiab]） AND（"polycystic ovary syndrome"[Mesh] OR "polycystic ovarian syndrome"[tiab] OR "PCOS"[tiab] OR "Rotterdam criteria"[tiab]） | 610 |
| #6 | （"fertilization in vitro"[Mesh] OR "IVF"[tiab] OR " sperm injections, intracytoplasmic"[Mesh] OR "ICSI"[tiab] OR "assisted reproductive technology"[tiab]） AND （"pregnancy outcome"[Mesh] OR "pregnancy rate"[tiab] OR "live birth"[tiab] OR "clinical pregnancy"[tiab] OR "miscarriage"[tiab] OR "abortion, spontaneous"[Mesh]) | 5,599 |
| #7 | （"anti-mullerian hormone"[Mesh] OR "AMH"[tiab] OR "anti mullerian hormone"[tiab] OR "müllerian-inhibiting substance"[tiab]） AND（"polycystic ovary syndrome"[Mesh] OR "polycystic ovarian syndrome"[tiab] OR "PCOS"[tiab] OR "Rotterdam criteria"[tiab]） AND（"fertilization in vitro"[Mesh] OR "IVF"[tiab] OR " sperm injections, intracytoplasmic "[Mesh] OR "ICSI"[tiab] OR "assisted reproductive technology"[tiab]） | 119 |
| #8 | （"anti-mullerian hormone"[Mesh] OR "AMH"[tiab] OR "anti mullerian hormone"[tiab] OR "müllerian-inhibiting substance"[tiab]） AND（"polycystic ovary syndrome"[Mesh] OR "polycystic ovarian syndrome"[tiab] OR "PCOS"[tiab] OR "Rotterdam criteria"[tiab]） AND ("pregnancy outcome"[Mesh] OR "pregnancy rate"[tiab] OR "live birth"[tiab] OR "clinical pregnancy"[tiab] OR "miscarriage"[tiab] OR "abortion, spontaneous"[Mesh]) | 83 |
| #9 | （"anti-mullerian hormone"[Mesh] OR "AMH"[tiab] OR "anti mullerian hormone"[tiab] OR "müllerian-inhibiting substance"[tiab]） AND（"polycystic ovary syndrome"[Mesh] OR "polycystic ovarian syndrome"[tiab] OR "PCOS"[tiab] OR "Rotterdam criteria"[tiab]） AND（"fertilization in vitro"[Mesh] OR "IVF"[tiab] OR " sperm injections, intracytoplasmic "[Mesh] OR "ICSI"[tiab] OR "assisted reproductive technology"[tiab]） AND ("pregnancy outcome"[Mesh] OR "pregnancy rate"[tiab] OR "live birth"[tiab] OR "clinical pregnancy"[tiab] OR "miscarriage"[tiab] OR "abortion, spontaneous"[Mesh]) AND ("cohort studies"[Mesh] OR "case-control studies"[Mesh] OR "observational study"[tiab] OR "prospective study"[tiab] OR "retrospective study"[tiab]) | 30 |
