# Supplementary material for: Anti-Müllerian hormone levels and live birth rate in polycystic ovary syndrome patients undergoing assisted reproductive technology: a systematic review and meta-analysis
Source: Front Med (Lausanne). 2026 Jul 28;13:1859573. doi: 10.3389/fmed.2026.1859573 (PMC13457698; doi:10.3389/fmed.2026.1859573)
Supplement: Supplementary file 2 [file Table_2.docx]

Supplementary table 2. Risk of bias assessments according to Newcastle-Ottawa Scale.

| **Cohort studies** | **Selection** | | | | **Comparability** | **Outcome** | | | **Total**  **score** |
| --- | --- | --- | --- | --- | --- | --- | --- | --- | --- |
|  | **Representativeness of the expose cohort** | **Selection of the non-exposed cohort** | **Ascertainment of exposure** | **Outcome of interest was not present at the start of the study** | **Comparability of cohorts on the basis of the design or analysis** | **Assessment of outcome** | **Was follow-up long enough for outcomes to occur?** | **Adequacy of follow up of cohorts** |  |
| K.S. Acharya 2022 | * | * | * |  | ** | * | * | * | 8 |
| C.Arkfeld 2022 | * | * | * |  | ** | * | * |  | 7 |
| A.A. Mohamed 2022 | * | * | * |  | ** | * | * |  | 7 |
| A.S. Komorowski 2024 | * | * | * |  | * | * | * |  | 6 |
| E. Vale-Fernandes 2023 | * | * | * |  | * | * | * |  | 6 |
| H.Huang 2023 | * | * | * |  | ** | * | * |  | 7 |
| N.Su 2023 | * | * |  | * | ** | * | * |  | 7 |
| R. Tal 2020 | * | * | * |  | ** | * |  | * | 7 |
| S.Liu 2022 | * | * | * |  | ** | * | * |  | 7 |
| X. Liu 2022 | * | * |  | * | ** | * | * | * | 8 |
| Y.Guo 2021 | * | * |  |  | ** | * | * |  | 6 |
| Y.Hou 2023 | * | * | * |  | ** | * | * | * | 8 |
| F.Zhao 2025 | * | * |  | * | * |  | * | * | 6 |
| Tongjie Li 2025 | * | * | * |  | ** | * | * | * | 8 |
